# Supplementary material for: Maximum entropy and population heterogeneity in continuous cell cultures
Source: PLoS Comput Biol. 2019 Feb 27;15(2):e1006823. doi: 10.1371/journal.pcbi.1006823 (PMC6411232; doi:10.1371/journal.pcbi.1006823)
Supplement: S1 Appendix — Additional mathematical derivations. (PDF) [file pcbi.1006823.s001.pdf]

Maximum entropy and population heterogeneity in continuous cell cultures. Appendix

Jorge Fernandez-de-Cossio-Diaz*[1,2] and Roberto Mulet†[1]

[1]Group of Complex Systems and Statistical Physics, Department of Theoretical Physics, University of Havana, Physics Faculty, Cuba
[2]Systems Biology Department, Center of Molecular Immunology, Havana, Cuba

November 27, 2018

*cossio@cim.sld.cu
†mulet@fisica.uh.cu

The expectation propagation approximation

At $\beta = 0$, the probability distribution of reaction fluxes in the population of cells is the uniform measure in the polytope of feasible states $\mathcal{P}$. We can represent this distribution by a truncated multivariate Gaussian [1],

$$\mathrm{e}^{-\frac{\eta}{2}(\mathbf{S}\mathbf{v}-\mathbf{b})^2}\prod_n \psi_n(v_n) \tag{1}$$

where $\psi_n(v_n) = 1$ if $\mathrm{lb}_n \leq v_n \leq \mathrm{ub}_n$ and $\psi_n(v_n) = 0$ otherwise, and for a large enough value of the parameter η. The truncation makes the marginals of this distribution hard to compute, so we instead use the approximate multivariate Gaussian,

$$Q_0(\mathbf{v}) \propto \mathrm{e}^{-\frac{\eta}{2}(\mathbf{S}\mathbf{v}-\mathbf{b})^2}\prod_n \phi_n(v_n) \propto \mathrm{e}^{-\frac{1}{2}(\mathbf{v}-\bar{\mathbf{v}})^T\mathbf{\Sigma}^{-1}(\mathbf{v}-\bar{\mathbf{v}})} \tag{2}$$

where $\phi_n(v_n) \propto \mathrm{e}^{-\frac{1}{2}(v_n-a_n)/d_n}$ are univariate Gaussians intended to approximate the truncation factors $\psi_n(v_n)$, and

$$\mathbf{\Sigma}^{-1} = \eta\mathbf{S}^T\mathbf{S} + \mathbf{D} \tag{3}$$

$$\bar{\mathbf{v}} = \mathbf{\Sigma}\left(\eta\mathbf{S}^T\mathbf{b} + \mathbf{D}\mathbf{a}\right) \tag{4}$$

and $\mathbf{D}$ is a diagonal matrix with entries $D_{nn} = 1/d_n$. The $2N$ parameters a_n, d_n are obtained from the $2N$ moment-matching conditions [2],

$$\langle v_n\rangle_{Q_0} = \langle v_n\rangle_{Q_0^{(n)}}, \quad \left\langle v_n^2\right\rangle_{Q_0} = \left\langle v_n^2\right\rangle_{Q_0^{(n)}} \tag{5}$$

where

$$Q_0^{(n)}(\mathbf{v}) = Q_0(\mathbf{v})\frac{\psi_n(v_n)}{\phi_n(v_n)} \propto \mathrm{e}^{-\frac{1}{2}\left(\mathbf{v}-\bar{\mathbf{v}}^{(n)}\right)^T\left(\mathbf{\Sigma}^{(n)}\right)^{-1}\left(\mathbf{v}-\bar{\mathbf{v}}^{(n)}\right)}\psi_n(v_n) \tag{6}$$

$$\left(\mathbf{\Sigma}^{(n)}\right)^{-1} = \eta\mathbf{S}^T\mathbf{S} + \mathbf{D}^{(n)} \tag{7}$$

$$\bar{\mathbf{v}}^{(n)} = \mathbf{\Sigma}^{(n)}\left(\eta\mathbf{S}^T\mathbf{b} + \mathbf{D}^{(n)}\mathbf{a}\right) \tag{8}$$

Upon convergence, the EP site distributions $\phi_n(v_n)$ are an approximation of the real site distributions $\psi_n(v_n)$. To incorporate the exponential selection prior, we define:

$$Q_{\boldsymbol{\beta}}(\mathbf{v}) = \mathrm{e}^{\boldsymbol{\beta}'\mathbf{v}}\mathrm{e}^{-\frac{\eta}{2}(\mathbf{S}\mathbf{v}-\mathbf{b})^2}\prod_n \phi_n(v_n) \propto \mathrm{e}^{-\frac{1}{2}(\mathbf{v}-\bar{\mathbf{w}})^T\mathbf{\Sigma}^{-1}(\mathbf{v}-\bar{\mathbf{w}})} \tag{9}$$

$$Q_{\boldsymbol{\beta}}^{(n)}(\mathbf{v}) = Q_{\boldsymbol{\beta}}(\mathbf{v}) \frac{\psi_n(v_n)}{\phi_n(v_n)} \propto \mathrm{e}^{-\frac{1}{2}\left(\mathbf{v}-\bar{\mathbf{w}}^{(n)}\right)^T \left(\boldsymbol{\Sigma}^{(n)}\right)^{-1} \left(\mathbf{v}-\bar{\mathbf{w}}^{(n)}\right)} \psi_n(v_n) \tag{10}$$

where $\bar{\mathbf{w}} = \bar{\mathbf{v}} + \boldsymbol{\Sigma}\boldsymbol{\beta}$ and $\bar{\mathbf{w}}^{(n)} = \bar{\mathbf{v}}^{(n)} + \boldsymbol{\Sigma}^{(n)}\boldsymbol{\beta}$. The vector $\boldsymbol{\beta}$ contains the selection coefficients of each reaction flux in the network. In the main text, this vector has a non-zero component in the biomass synthesis reaction, while all other components are zero. Then we estimate the marginal distribution of flux v_n under the exponential selection prior as a truncated univariate Gaussian:

$$P_{\boldsymbol{\beta}}(v_n) \approx \int Q_{\boldsymbol{\beta}}^{(n)}(\mathbf{v}) \, \mathrm{d}\mathbf{v}_{\setminus n} \propto \mathrm{e}^{-\frac{1}{2}\left(v_n - \bar{w}_n^{(n)}\right)^2 / \boldsymbol{\Sigma}_{nn}^{(n)}} \psi_n(v_n) \tag{11}$$

Derivation of Equation 13 from Michaelis-Menten kinetics

An alternative uptake bound used in the literature is:

$$u_i \leq \frac{V_i s_i}{K_i + s_i} \tag{12}$$

where V_i is the maximum uptake rate of metabolite i and K_i is the Michaelis-Menten constant. Equation (13) in the main text can be derived as an approximation to equation (12) [3]. If a substrate is available in excess ($s_i \gg K_i$), this bound simplifies to $u_i \leq V_i$. In rich media at low cell densities this is the relevant regime. At higher cell-densities, substrates reach low levels ($s_i \ll K_i$), and the bound is well approximated by $u_i \leq s_i V_i / K_i$. Employing the steady state metabolite concentration from Eq. (12) in the main text, $s_i = c_i - \langle u_i \rangle \xi$, where $\langle u_i \rangle$ is the average uptake rate of metabolite i in the population of cells. Making the mean-field approximation $u_i \approx \langle u_i \rangle$, we obtain $u_i \leq (c_i - u_i \xi) V_i / K_i$, or

$$u_i \leq \frac{c_i V_i / K_i}{1 + \xi V_i / X_i} \tag{13}$$

For high cell densities $\xi V_i / K_i \gg 1$, and the inequality simplifies to $u_i \leq c_i / \xi$. Combining the bounds obtained in both regimes leads to Equation (13) in the main text. Note that, compared to Equation (12), this approximated bound contains one parameter (since it does not depend on K_i), and it simplifies mathematical derivations.

Stochastic model

To gain some intuition for the metabolic distribution described in the previous section, we conceived a simple stochastic model of a population of cells evolving in metabolic space. As in the main text, a given value of ξ determines a polytope $\mathcal{P}_\xi$ of feasible metabolic states. We start the simulation with N cells placed randomly inside $\mathcal{P}_\xi$. Let $\vec{v}_\ell \in \mathcal{P}_\xi$ be the metabolic state of cell ℓ, for $\ell = 1, \ldots, N$. At each step, we select a random cell for reproduction. Cell ℓ has a probability proportional to μ_ℓ of being selected for reproduction, where $\mu_\ell\left(\vec{v}_\ell\right)$ is its growth rate. The selected cell replicates to generate a new cell. With a probability ϵ, the newborn has a random metabolic state; thus ϵ is a probability of "mutation" in this model. Otherwise, with a probability $1-\epsilon$ the newborn has the same growth rate as the mother cell, in which case its metabolic state is a random choice among all metabolic states consistent with this growth rate. Moreover, whenever a cell reproduces, we also select a random cell from the population and eliminate it. This way the total number of cells, N, is kept constant.

Assuming that N is sufficiently large, this simulation will converge after many steps to a stationary population where average statistics $\sum_\ell f\left(\vec{v}_\ell\right)/N$ of arbitrary functions $f\left(\vec{v}\right)$ of the flux vector will be approximately constant. In particular, we obtain $\langle u_i \rangle$, the uptake rates, and from these, the metabolite concentrations $s_i = c_i - \langle u_i \rangle \xi$.

We simulated populations of 10000 cells, for different values of ξ and ϵ. In each case we recorded the steady state distribution of cells in the polytope $\mathcal{P}_\xi$. In particular the simulation resulted in a steady average flux of ATP production, $\langle v_{atp} \rangle$. We then compare the distribution of cells in the polytope from the simulation with the MaxEnt distribution obtained at the same value of ξ, where β is fixed by demanding that the average flux of ATP is the same as in the simulation. Due to the low dimensionality of this simple metabolic model, the resulting equation:

$$\frac{\int_{\mathcal{P}_\xi} \mathrm{e}^{-\beta y v_{atp}} v_{atp} \mathrm{d}v_{atp} \mathrm{d}v_g}{\int_{\mathcal{P}_\xi} \mathrm{e}^{-\beta y v_{atp}} \mathrm{d}v_{atp} \mathrm{d}v_g} = \langle v_{atp} \rangle \tag{14}$$

can be solved numerically for β.

The comparison between the population histograms in steady state and the coresponding MaxEnt distribution are shown in Fig. 1, for some representative values of the parameters ξ, ϵ. Here the plot represents the marginal flux distribution $P_\xi\left(v_{atp}\right)$ in the population. The corresponding root β of Eq. (14) is also shown.

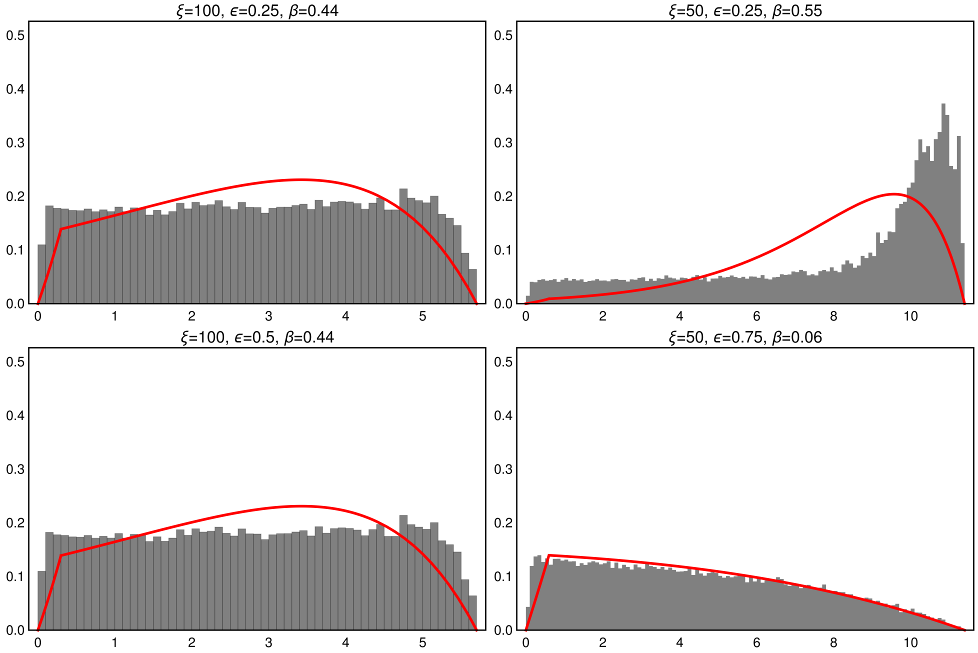

Figure 1: Comparison of MaxEnt distribution (red) and stationary distribtion from the stochastic model (gray bars) for v_{atp}.

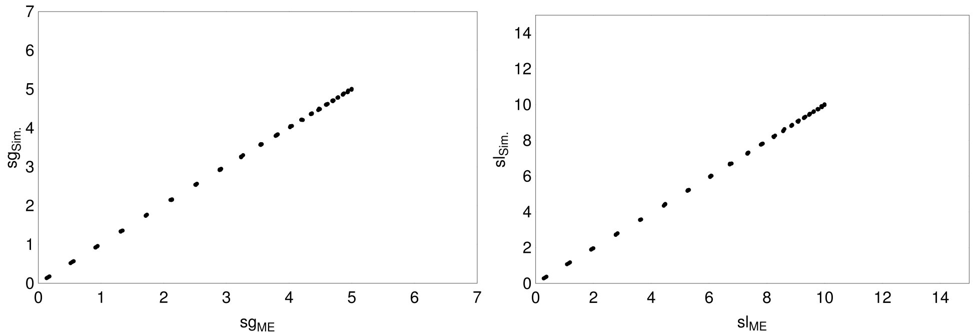

Figure 2: Glucose (s_g) and lactate (s_l) concentrations, inferred from MaxEnt (ME) compared to the steady state value reached in the simulations (Sim.).

Finally, the metabolite concentrations are found from $s_i = c_i - \xi \langle u_i \rangle$, where c_i is the feed concentration of metabolite i and $\langle u_i \rangle$ the average uptake in the population. We compute $\langle u_i \rangle$ from the steady state distribution, and from the MaxEnt formalism. Note that $\langle v_{atp} \rangle_{\mathrm{ME}} = \langle v_{atp} \rangle_{\mathrm{Sim.}}$ by definition, where ME denotes the MaxEnt estimate and Sim. the simulation result. On the other hand, $\langle v_g \rangle_{\mathrm{ME}}$ and $\langle v_g \rangle_{\mathrm{Sim.}}$ can be different in principle. Figure 2 shows the comparison between the inferred values (using the MaxEnt estimate, ME) and the simulation results.

Sensitivity to variations in crowding coefficients

To assess the sensitivity of our qualitative results in the CHO-K1 genome-scale model to variations in the crowding coefficients, we repeated simulations after randomly perturbing these coefficients by as much as 25%. Comparing the following figures to Figures 6 and 7 in the text reveals no significant changes.

Reduced CHO-K1 model

The reduced CHO-K1 model is provided in supplementary materials in *.jld2* format (https://github.com/JuliaIO/JLD2.jl). The file contains three variables that represent the reduced model: *nz_S* is the sotoichiometric matrix, *nz_mets* the table of metabolites and *nz_rxns* the table of reactions.

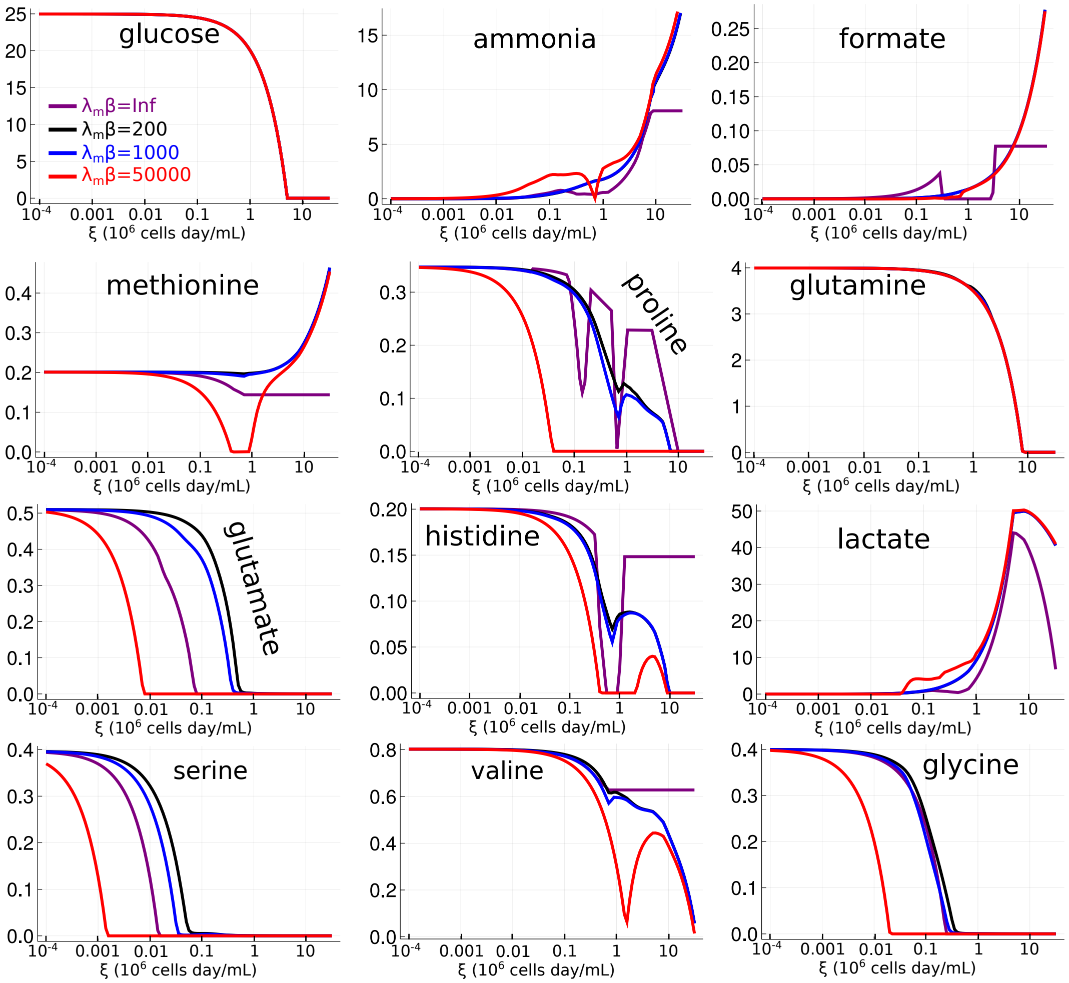

Figure 3: **Steady state metabolite concentrations as functions of ξ for the CHO-K1 model.** Steady state concentrations of selected metabolites as functions of ξ, for the simulations of the CHO-K1 metabolic network, after 25% relative perturbations in crowding coefficients. Representative values of β are plotted. The black line corresponds to the $\beta \to \infty$ limit.

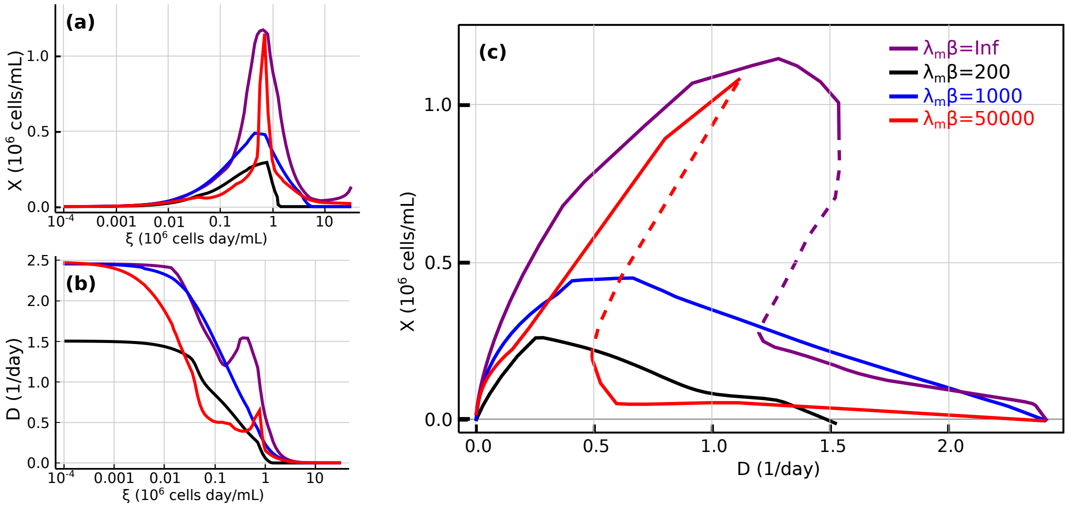

Figure 4: **Dilution rate versus cell density in steady state of the CHO-K1 metabolic model.** Different curves correspond to different levels of heterogeneity. Discontinuous line indicates unstable steady states.

References

[1] Alfredo Braunstein, Anna Paola Muntoni, and Andrea Pagnani. An analytic approximation of the feasible space of metabolic networks. *Nature Communications*, 8:14915, April 2017.

[2] Thomas P. Minka. Expectation Propagation for Approximate Bayesian Inference. In *Proceedings of the Seventeenth Conference on Uncertainty in Artificial Intelligence*, UAI'01, pages 362–369, San Francisco, CA, USA, 2001. Morgan Kaufmann Publishers Inc.

[3] Jorge Fernandez-de Cossio-Diaz, Kalet Leon, and Roberto Mulet. Characterizing steady states of genome-scale metabolic networks in continuous cell cultures. *PLoS Computational Biology*, 13(11):e1005835, 2017.
